# Assessment of Soil Features on the Growth of Environmental Nontuberculous Mycobacterial Isolates from Hawai'i

**DOI:** 10.1128/AEM.00121-20

**Published:** 2020-10-15

**Authors:** Cody M. Glickman, Ravleen Virdi, Nabeeh A. Hasan, L. Elaine Epperson, Leeza Brown, Stephanie N. Dawrs, James L. Crooks, Edward D. Chan, Michael Strong, Stephen T. Nelson, Jennifer R. Honda

**Affiliations:** a Center for Genes, Environment and Health, National Jewish Health, Denver, Colorado, USA; b Department of Geological Sciences, Brigham Young University, Provo, Utah, USA; c Division of Biostatistics and Bioinformatics, National Jewish Health, Denver, Colorado, USA; d Medicine and Academic Affairs, National Jewish Health, Denver, Colorado, USA; e Division of Pulmonary Sciences and Critical Care Medicine, University of Colorado Anschutz Medical Campus, Aurora, Colorado, USA; f Department of Medicine, Rocky Mountain Regional Veterans Affairs Medical Center, Denver, Colorado, USA; University of Tennessee at Knoxville

**Keywords:** nontuberculous mycobacteria, soil minerals, Hawai’i

## Abstract

Environmental nontuberculous mycobacteria (NTM), with the potential to cause opportunistic lung infections, can reside in soil. This might be particularly relevant in Hawai’i, a geographic hot spot for NTM infections and whose soil composition differs from many other areas of the world. Soil components are likely to contribute to NTM prevalence in certain niches as food sources or attachment scaffolds, but the particular types of soils, clays, and minerals that impact NTM growth are not well-defined. Hawai’i soil and chemically weathered rock (saprolite) samples were examined to characterize the microbiome and quantify 11 mineralogical features as well as soil pH. Machine learning methods were applied to identify important soil features influencing the presence of NTM. Next, these features were directly tested *in vitro* by incubating synthetic clays and minerals in the presence of Mycobacteroides abscessus and Mycobacterium chimaera isolates recovered from the Hawai'i environment, and changes in bacterial growth were determined. Of the components examined, synthetic gibbsite, a mineral form of aluminum hydroxide, inhibited the growth of both M. abscessus and M. chimaera, while other minerals tested showed differential effects on each species. For example, M. abscessus (but not M. chimaera) growth was significantly higher in the presence of hematite, an iron oxide mineral. In contrast, M. chimaera (but not M. abscessus) counts were significantly reduced in the presence of birnessite, a manganese-containing mineral. These studies shed new light on the mineralogic features that promote or inhibit the presence of Hawai’i NTM in Hawai’i soil.

**IMPORTANCE** Globally and in the United States, the prevalence of NTM pulmonary disease—a potentially life-threatening but underdiagnosed chronic illness—is prominently rising. While NTM are ubiquitous in the environment, including in soil, the specific soil components that promote or inhibit NTM growth have not been elucidated. We hypothesized that NTM culture-positive soil contains minerals that promote NTM growth *in vitro*. Because Hawai’i is a hot spot for NTM and a unique geographic archipelago, we examined the composition of Hawai’i soil and identified individual clay, iron, and manganese minerals associated with NTM. Next, individual components were evaluated for their ability to directly modulate NTM growth in culture. In general, gibbsite and some manganese oxides were shown to decrease NTM, whereas iron-containing minerals were associated with higher NTM counts. These data provide new information to guide future analyses of soil-associated factors impacting persistence of these soil bacteria.

## Abstract (precis)

Globally and in the United States, the prevalence of NTM pulmonary disease—a potentially life-threatening but underdiagnosed chronic illness—is prominently rising. While NTM are ubiquitous in the environment, including in soil, the specific soil components that promote or inhibit NTM growth have not been elucidated. We hypothesized that NTM culture-positive soil contains minerals that promote NTM growth *in vitro*. Because Hawai’i is a hot spot for NTM and a unique geographic archipelago, we examined the composition of Hawai’i soil and identified individual clay, iron, and manganese minerals associated with NTM. Next, individual components were evaluated for their ability to directly modulate NTM growth in culture. In general, gibbsite and some manganese oxides were shown to decrease NTM, whereas iron-containing minerals were associated with higher NTM counts. These data provide new information to guide future analyses of soil-associated factors impacting persistence of these soil bacteria.

## INTRODUCTION

Natural and human-made environments harbor potentially disease-causing species of nontuberculous mycobacteria (NTM) (1). The NTM species responsible for human lung infections are thought to be influenced by the specific environmental source exposures and the NTM species diversity within these environmental niches. While water-associated biofilms contain potentially disease-causing NTM, a variety of NTM species have also been discovered in soil (2–4). Prior studies have shown that potting soil can be a reservoir for clinically relevant mycobacteria (4). In Japan, residential soil from patients with pulmonary NTM infections were demonstrated to harbor NTM that were genetically related to patients’ respiratory NTM isolates, and that soil was a source of the patients’ polyclonal and mixed Mycobacterium avium complex infections (5, 6). In the United States, Hawai’i has the highest prevalence of NTM lung infections, with almost four times higher NTM infection rates than the national average in a survey among older adults (7). In prior work (8), we reported the presence of clinically relevant slow-growing mycobacteria (SGM), including Mycobacterium chimaera, Mycobacterium marseillense, and Mycobacterium intracellulare, in Hawai’i soil samples, in addition to rapid-growing mycobacteria (RGM), including Mycolicibacterium septicum and Mycolicibacterium alvei.

The breadth of NTM species diversity in soil is likely driven by the proportion and composition of minerals and nutrients in that particular soil sample. For example, larger amounts of metals such as copper and cations such as sodium have been shown to be significant predictors for NTM infection in the United States. (9). Prior studies from Queensland, Australia, have shown soil containing nutrients such as nitrate or having low pH predicted the presence of RGM, including Mycolicibacterium fortuitum and Mycobacteroides chelonae (10). Yet soil components such as natural rock, sand, and clay may also impact NTM presence and diversity. A study by Lipner et al. reported increasing clay concentrations as protective against NTM, while increasing silt concentrations were associated with NTM infection (11). In this same study and another, a higher manganese concentration was associated with disease prevalence (9, 11). Thus, variable soil characteristics and components may either inhibit or promote NTM growth in soil.

In the current study, we performed microbiome and mineral/chemical analyses on a set of Hawai’i soil samples and tested the impact of particular clays and chemicals on the *in vitro* growth of native NTM species recovered from the Hawai’i environment. Since almost all of the rock underlying Hawai’i ecosystems is oceanic basalt comprised of volcanic rock with limited variations in composition (12), the characteristics associated with the presence of NTM in Hawai’i soil may significantly vary from what has been described so far.

## RESULTS

### Less diverse microbiome in NTM culture-positive soil samples.

Of the total soil samples from this study, a subset (*n* = 18, comprised of 8 NTM culture positive and 10 NTM culture negative) were subjected to exploratory microbiome analyses. Linear modeling of mycobacterium genus counts against pH and culture status revealed no significant relationships (*F* = 1.007 and *P* = 0.330 and *F* = 1.119 and *P* = 0.306, respectively). The Shannon diversity index was used to compare species richness between NTM culture-positive and NTM culture-negative groups. The Shannon diversity values between NTM culture groups showed a trend toward significance, with NTM culture-positive samples having a lower overall diversity than NTM culture-negative samples (Fig. S4A in the supplemental material). Taxonomic analysis revealed the phylum *Firmicutes* was significantly enriched in NTM culture-positive samples (Fig. S4B) and the phylum composition varied by culture status (Fig. S4C). Similar trends in Shannon diversity and overall phyla abundance were also observed when the data were stratified by mycobacteria genus counts (Fig. S5A and B).

### NTM recovery is not driven by soil pH.

**FIG 1:**
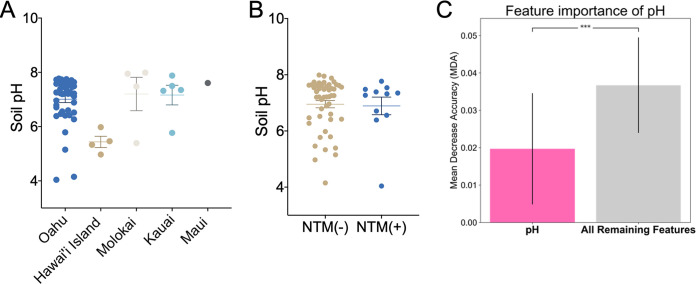
NTM culture results are not related to soil pH. (A) pH was measured from soil samples collected from Oahu (*n* = 46), Hawai’i Island (*n* = 4), Molokai (*n* = 4), Kauai (*n* = 5), and Maui (*n* = 1). pH value distributions were plotted by island and tested for differences (one-way ANOVA; *, *P = *0.01). (B) Soil samples were stratified by NTM culture status, and all pH values are plotted. NTM culture-negative samples, pH mean = 6.95 (*n* = 49); NTM culture-positive samples, pH mean = 6.89 (*n* = 11) (one-way ANOVA; not significant; *P = *0.85). (C) Feature importance is defined by mean decrease in accuracy (MDA) after 1,000 iterations of a classifier while shuffling the feature values. A higher MDA is associated with an important feature in the model. All remaining features is an average of the importance and variation among features other than pH. The importance of pH is lower than the average of all remaining features (0.0197 ± 0.0145 versus 0.0367 ± 0.0128; ***, *P < *0.0001).

Soil was subjected to pH analyses. Overall, there was a statistically significant difference in soil pH among the individual islands. Specifically, of the five islands examined, the pH of Hawai’i Island soil was more acidic (mean pH = 5.4) than the soil pH of 7.1 to 7.6 of the other four islands (Fig. 1A). However, pH did not significantly vary when the data were stratified by NTM culture results (Fig. 1B). Compared to an average of all other features in the data set, the importance of pH as a feature had a lower mean decrease in accuracy (Fig. 1C).

### Gibbsite, a clay mineral, inhibits both M. abscessus and M. chimaera *in vitro*.

**FIG 2:**
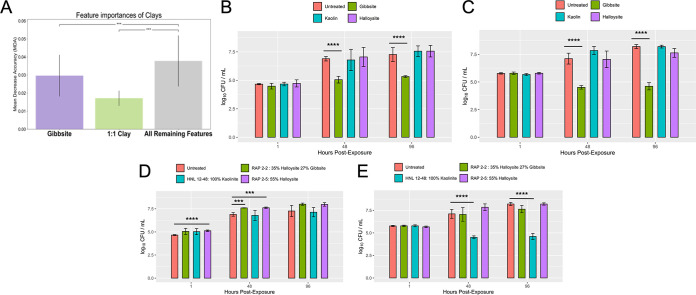
Impact of clay minerals on the *in vitro* growth of native Hawai’i environmental NTM isolates. (A) Distribution of clay mineral mean decrease in accuracy across 1,000 iterations of shuffling. The importance of gibbsite is less than the average of the remaining features (0.0302 ± 0.0117 versus 0.0377 ± 0.0141). However, the importance of gibbsite is greater than 1:1 clay (0.0302 ± 0.0117 versus 0.0172 ± 0.0041). (B) *In vitro* growth of M. abscessus in the presence of synthetic gibbsite, kaolin, and halloysite. (C) *In vitro* growth of M. chimaera in the presence of synthetic gibbsite, kaolin, or halloysite. (D) *In vitro* growth of M. abscessus in the presence of Hawai’i soil. (E) *In vitro* growth of M. chimaera in the presence of Hawai’i soil. ***, *P* < 0.001; ****, *P* < 0.0001.

Exploratory feature importance selection was then performed against the full data set to elucidate possible clay characteristics that correlate to the presence of NTM. Based on our feature prediction models, gibbsite (a clay mineral) and 1:1 clays (a group of structurally related minerals where the fundamental building block consists of a sheet of silicate tetrahedra bonded to a layer of Al-O-OH or Mg-O-OH octahedra, which includes kaolinite and halloysite, the most common 1:1 clays in Hawai’i) (13, 14) were predicted to be less important compared to all other features in the data set (Fig. 2A). Alternatively, based again on our machine learning models, NTM culture-negative samples were predicted to have more gibbsite and 1:1 clays when stratified by culture status (Fig. S6A). To directly test these hypotheses, gibbsite and the clays kaolin and halloysite were incubated separately in the presence of NTM. Synthetic gibbsite significantly inhibited the growth of both M. abscessus and M. chimaera *in vitro* (Fig. 2B and C), as well as M. avium, at 48 h (Fig. S7A) compared to the untreated controls. The growth of M. abscessus and M. chimaera was not significantly altered by exposure to kaolin or halloysite (Fig. 2B and C), but halloysite significantly facilitated the growth of M. avium at 96 h (Fig. S7A).

To investigate whether the aforementioned results could be replicated using actual soil samples, three Hawai’i soil samples were identified to contain (i) 100% kaolinite (Honolulu [HNL] sample 12-48), (ii) 35% halloysite/27% gibbsite (rappelling sample 2-2 [RAP2-2]), or (iii) 55% halloysite (RAP2-5). Similar to Fig. 2B, the growth of M. abscessus was not impacted when cocultured with HNL 12-48, a soil comprised of 100% kaolinite (Fig. 2D); in contrast, significantly less M. chimaera was recovered (Fig. 2E). The inhibitory effect of gibbsite on NTM growth was lost when incubated with soil containing both gibbsite and halloysite (Fig. 2D and E). M. abscessus showed significantly higher growth early after exposure to RAP2-5 (1 and 48 h) and RAP2-2 soil samples (48 h); however, incubation with these soils did not affect M. chimaera growth *in vitro* compared to the bacteria alone group (untreated) (Fig. 2D and E).

### Iron minerals significantly increase NTM growth *in vitro*.

**FIG 3:**
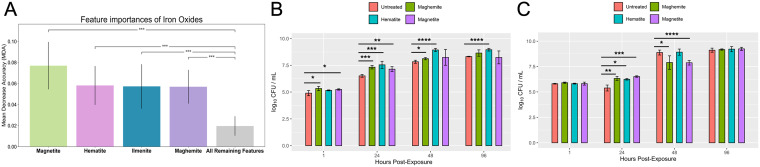
Impact of iron minerals on the *in vitro* growth of native Hawai’i environmental NTM isolates. (A) Distribution of iron oxide mean decrease in accuracy across 1,000 iterations of shuffling. The lowest iron oxide mineral (maghemite) is greater than the average of all remaining features, suggesting iron oxide minerals are important for NTM growth (0.0569 ± 0.0160 versus 0.0196 ± 0.0092). Magnetite is of greater importance than maghemite (0.0770 ± 0.0226 versus 0.0569 ± 0.0160) or hematite (0.0770 ± 0.0226 versus 0.0581 ± 0.0185). (B) *In vitro* growth of M. abscessus in the presence of synthetic maghemite, magnetite, and hematite. (C) *In vitro* growth of M. chimaera in the presence of synthetic maghemite, magnetite, and hematite. *, *P* < 0.05; **, *P* < 0.01; ***, *P* < 0.001; ****, *P* < 0.0001.

Based on our feature prediction modeling, iron oxide minerals such as maghemite, hematite, and magnetite are posited to be of greater importance than the combination of all remaining features (Fig. 3A). To estimate the directionality of maghemite, hematite, and magnetite to NTM growth, the amounts of these iron oxides in each soil sample were plotted against NTM culture status, predicting more hematite and maghemite in NTM-positive cultures (Fig. S6B). Tested *in vitro*, the growth of M. abscessus was generally significantly enhanced in the presence of hematite and maghemite compared to the untreated control (Fig. 3B). While greater counts of M. chimaera were observed for all iron oxides tested at the 24-h mark than the untreated control (Fig. 3C), growth decreased at the 48-h time point in the samples incubated with maghemite and magnetite (Fig. 3C). However, CFU abundance in the samples was equivalent by the 96-h time point for all M. chimaera samples. Similar to M. abscessus, significantly more M. avium was observed when incubated with hematite (Fig. S7B).

### Manganese minerals show varied effects on NTM growth *in vitro*.

**FIG 4:**
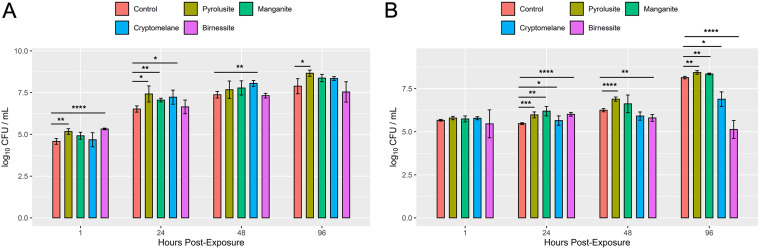
Impact of a manganese compound on the *in vitro* growth of native Hawai’i environmental NTM isolates. *In vitro* growth of M. abscessus (A) or M. chimaera (B) in the presence of synthetic manganese minerals. *, *P* < 0.05; **, *P* < 0.01; ***, *P* < 0.001; ****, *P* < 0.0001.

Because soil manganese has been associated with a lower risk for NTM infections (7, 9), the effects of manganese minerals on NTM growth were tested. M. abscessus, M. chimaera, and M. avium were incubated in the presence of manganese minerals, including synthetic pyrolusite, manganite, cryptomelane, and birnessite. Results were varied. In general, the growth of all NTM tested was significantly higher when cultured in the presence of the manganese oxide pyrolusite (Fig. 4A and B; Fig. S7C). While the growth of M. abscessus was also higher in the presence of cryptomelane (24 and 48 h), M. chimaera growth was significantly inhibited by the 96-h time point. Less M. chimaera was also observed in the presence of the manganese oxide mineral birnessite. Of importance, birnessite also significantly inhibited the growth of M. avium (Fig. S7C) while showing little effect on M. abscessus viability.

### Pictorial of NTM attachment to mineral surfaces.

**FIG 5:**
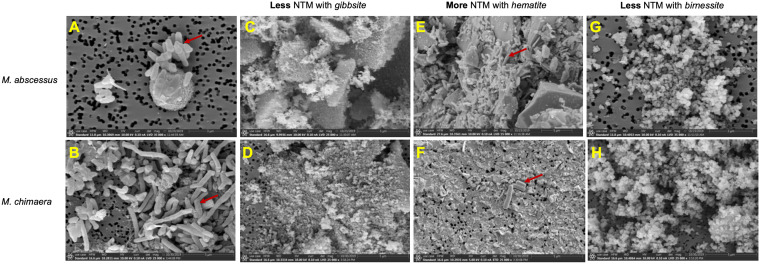
Scanning electron microscope images of environmental NTM isolates grown in the presence of gibbsite, hematite, and birnessite. M. abscessus (3 μm) (A) and M. chimaera (5 μm) (B) in the absence of soil minerals. M. abscessus (C) and M. chimaera (D) in the presence of gibbsite. M. abscessus (E) and M. chimaera (F) in the presence of hematite. M. abscessus (G) and M. chimaera (H) in the presence of birnessite. Red arrows indicate the NTM bacilli.

*In vitro* assays demonstrated more numbers of M. abscessus and M. chimaera in the presence of hematite, whereas gibbsite and birnessite significantly inhibited the growth of NTM. Scanning electron microscopy (SEM) images show M. abscessus (Fig. 5A) and M. chimaera (Fig. 5B) alone and in association with hematite (Fig. 5E and F), whereas no bacilli are seen in the presence of gibbsite (Fig. 5C and D) and birnessite (Fig. 5G and H).

## DISCUSSION

Infections due to NTM are a growing clinical concern across the United States and many parts of the world due to their increasing prevalence and their recalcitrant nature to current chemotherapeutic treatments. It is widely recognized that environmental exposures contribute to NTM acquisition. While not as widely sampled or well characterized as water-associated biofilms, NTM occupy soil niches globally. Because 80 to 90% of microbes in soil are attached to solid surfaces, understanding the specific components of soil that contribute to NTM growth and maintenance in a geographic focal point for infection like Hawai’i is imperative (15). In this study, we performed microbiome and mineralogic studies and applied permuted feature importance approaches to predict soil components associated with NTM. We then tested the ability of these soil components to directly modulate the growth of native NTM isolates from Hawai’i *in vitro* and used high-powered microscopy to capture the capability of NTM to bind to these components.

The microbiome study demonstrated a trend toward lower alpha diversity in the NTM culture-positive samples, suggesting reduced richness of species compared to the NTM culture-negative samples (Fig. S4A). If the trend of lower alpha diversity in NTM culture-positive samples remains true when the sample size increases, this metric could potentially become a useful feature to predict NTM presence or absence in soil. Alpha diversity may also be linked to soil composition and competition for resources.

A variety of soil factors, or a combination of factors, could contribute to the presence or absence of environmental NTM. When analyzed as a single factor, soil pH was not found to be a significant driver for NTM diversity in this study (Fig. 1B), despite NTM showing a preference for acidic environmental conditions (pH 3 to 5) (e.g., acidic, brown water swamps; fulvic and humic acids; and peat-rich potting soils) (16–18). Most soils in Hawai’i have pH ranges from 4 to 8, but most are acidic due to the warm temperatures and high rainfall, leading to elevated pCO_2_ values in the atmosphere. However, a primary driver of low pH in deeply weathered soils is the lack of base cations. Because we observed Hawai’i Island soil as more acidic than the other islands examined, future studies should further elucidate the role of soil pH to NTM growth.

A primary aim of this work was to determine important soil mineralogical features associated with NTM culture status by using machine learning tools to identify important features and then validating the impact of these minerals on NTM growth *in vitro*. Overall, soil feature distributions did not correlate directly with *in vitro* NTM culture assays. This may be a result of the limited power and the unbalanced outcome groupings. Yet, by using feature importance measures, we were able to identify gibbsite as a possible modulator of NTM growth (Fig. 2A) and confirmed that alone, pure gibbsite significantly inhibits the growth of M. abscessus and M. chimaera (Fig. 2B and C). Gibbsite is one of the mineral forms of aluminum hydroxide that forms the weathered surfaces of clays. Prior work detailing the soil composition of the Colombian Amazon has shown aluminum in clay possesses antibacterial activity against other microorganisms, including Escherichia coli (19). While gibbsite is common in tropical soils (20), the amount of gibbsite or its interaction with other minerals may influence the presence or absence of NTM in Hawai’i soil. Beyond the examination of aluminum as a single factor, the combination of aluminum and iron has been shown to increase the production of reactive oxygen species in prokaryotes, which can cause cell death (21). Noteworthy of discussion, the inhibitory effect of gibbsite on NTM growth was lost when incubated with soil containing gibbsite and halloysite (Fig. 2D and E). It is possible that the surface chemistry and crystal size of pure gibbsite change when in a complex mixture such as soil containing other minerals (e.g., halloysite). Similar discrepancies were also observed for M. chimaera incubated with pure kaolin or kaolinite-containing soil. Incubation of M. chimaera with pure kaolin did not alter CFU counts at the time points tested (Fig. 2C); however, significantly less M. chimaera was recovered overtime when incubated in soil containing kaolinite (Fig. 2E). Besides kaolinite, it is possible the soil sample also contained other unidentified minerals or other factors that inhibited M. chimaera growth. It would be prudent to perform more detailed chemical analyses of these particular soils in the future.

The presence of iron in soil can promote NTM growth, and our feature predictions posit this is also true in Hawai’i soil (Fig. 3A). Our *in vitro* data indicate that not all iron oxide minerals such as maghemite, magnetite, and hematite impact the growth of NTM equally. For example, M. abscessus growth was facilitated in the presence of all iron oxides tested, particularly hematite (Fig. 3B), which was also associated with higher growth of M. avium (Fig. S7B) but with little impact on M. chimaera (Fig. 3C). Hematite has already been shown to promote the growth of soil-dwelling Pseudomonas mendocina by acting as an iron source *in vitro* (22). Because of the importance of iron equestration by NTM, it would be prudent for future work to dissect the role of M. abscessus, M. chimaera, and M. avium siderophores on iron sequestration from soil. Additionally, the impact of iron oxides on NTM biofilm development is an avenue for future study given the known effect of iron on the growth of M. smegmatis and M. tuberculosis biofilms (23, 24).

Manganese is an important minor element commonly found in basalts and other mafic rocks and is implicated as an inhibitory agent for NTM in soil. Although numerous manganese oxides and hydroxides (e.g., pyrolusite, manganite, and cryptomelane) have been identified, birnessite is one of the most common in soil (25). Birnessite demonstrated potent antibacterial activity against both SGM M. chimaera (Fig. 4B) and M. avium (Fig. S7C) but did not affect the growth of the RGM M. abscessus (Fig. 4A). M. chimaera growth was impaired in the presence of cryptomelane by the 96-h time point. Interestingly, the manganese oxide pyrolusite facilitated the growth of all three NTM species tested. In other studies, manganese oxide nanoparticles were found to exert antibacterial activity against Vibrio cholerae, *Shigella* species, and E. coli, and birnessite has been shown to inhibit pathogenic prions (26, 27). The role of manganese oxides/hydroxides in NTM growth in Hawaiian soils remains an open question. Additional work would be required to identify how manganese negatively or positively impacts NTM growth in the environment.

SEM images augment the culturing studies by illustrating the relationships between mineral substrates and NTM cells, although fixation and rinsing steps in mount preparations may not preserve a 1:1 relationship between cells and cell attachment versus abundances in culturing experiments. M. abscessus was seen in abundance attached to the surfaces of hematite grains and on the filter membrane of the mineral-free control culture, whereas it was not observed in the presence of birnessite and gibbsite (Fig. 5). Similar relationships were observed for M. chimaera. This species was found in the presence of hematite, although not in the relatively high proportions exhibited by M. abscessus, but was absent on gibbsite or birnessite (Fig. 5).

The absence of NTM in the presence of pure gibbsite (Fig. 2B and C) may be due to aluminum toxicity (19). In addition, gibbsite is very fine-grained, with crystallites <1 μm in diameter (Fig. 5), which may preclude attachment to a single grain. Similarly, individual birnessite grains are very small and unfavorable for attachment. Presumably, some aspects of the surface chemistry of birnessite may also contribute to the inhibition of M. chimaera and M. avium in soil.

This study introduced the possibility that transition metals and oxide features in soil influence NTM growth *in vitro*. Future work should elucidate the various mechanisms used by NTM to evade the toxicity of soil factors to promote extended survival in the environment. For example, RGM, including M. fortuitum and M. chelonae, have been shown to resist exposure to transitional metals such as mercury through actions of protective mercuric reductases and organomercurial lyases (28, 29). In addition, the type VII secretion systems (e.g., ESX-3) of environmental mycobacteria have been associated with iron acquisition via mycobactin, a secreted iron chelator that promotes survival (30). Finally, it is possible NTM also utilize other siderophores, chelating proteins such as calprotectins, or structures similar to “zincosomes” (zinc-holding compartments) produced by Mycobacterium tuberculosis, as multifaceted mechanisms to protect from soil toxicity including control of uptake, oxidation, sequestration inside the bacteria, and efflux of toxic soil materials (31).

**FIG 6:**
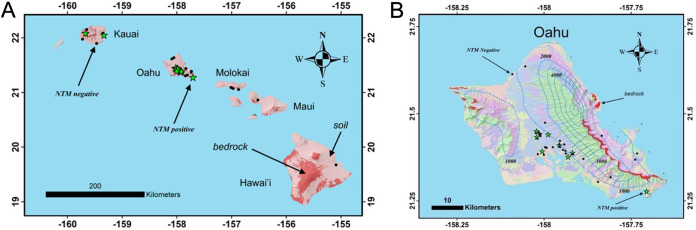
Hawai’i soil map. (A) Location of soil samples in the Hawaiian Islands, with NTM-positive (green stars) and NTM-negative soils (black dots) as indicated. (B) Map of Oahu indicating NTM-positive and -negative soils. Blue contours are mean annual rainfall (mm/yr). Red indicates the presence of bedrock at the surface and the other map colors represent various soil orders (48).

This study has some limitations. Soil samples were unequally collected from a limited number of sites across Oahu, Kauai, Hawai’i Island, Molokai, and Maui (Fig. 6). Collecting an increased number of soils that more widely and equally represent the different geographic areas across the islands would not only increase the limited sample size but will also provide a more complete study set to more robustly identify features that influence NTM growth. Increasing the number of NTM culture-positive samples with defined soil characteristics would also improve the balance of the data set and the overall feature selection performance. The addition of more samples would also increase the power of comparisons in the microbiome analysis. A single concentration of each mineral was used to compare across species and time points; however, the growth of NTM may be modulated with lower or larger amounts of compounds. Because soil is a complex mixture of many different components, we also cannot rule out the role of all other soil components (e.g., sodium, zinc, copper, and organic material) or other environmental factors such as rainfall and humidity in NTM growth and sustainability. Finally, because we were interested in studying M. abscessus and M. chimaera, clinically relevant NTM found in the lung, these experiments were performed at 37°C; however, soil temperature likely varies widely in the environment, and these results might change with lower incubation temperature.

In closing, this study is the first, to our knowledge, to characterize the soil composition in detail and relate that to NTM culture status. This study also identified important minerology features in Hawai’i soil using the application of machine learning tools, which were then validated *in vitro*. In addition, this study captured microscopy images of NTM binding to soil features. Because gibbsite and some of the manganese oxides were shown to decrease NTM growth and hematite, and pyrolusite promoted growth, it would be prudent to quantify these components and others in other soil samples globally in future work with subsequent translation of these findings to the presence or absence of clinically relevant NTM species in the environment.

## MATERIALS AND METHODS

### Soil samples and NTM isolates used in this study.

In 2012, 65 different soil samples were collected from locations across Oahu, Kauai, Hawai’i Island, Molokai, and Maui, and NTM culture diversity from these samples was previously reported (8). A subset of 55 samples was used for downstream processing due to missing data, and the samples’ collection sites are plotted in Fig. 6 based on global positioning system coordinates. The average rainfall of the sites ranged from less than 1,000 mm/year to 2,000 mm/year. Of note, this study did not consider soil types categorized by the USDA classification system. Of these soils, 13/65 (20%) were NTM culture positive. To assess the impact of soil minerals and components on NTM growth *in vitro*, two environmental Hawai’i NTM isolates were tested in the *in vitro* studies detailed herein, including 12-45-Sw-A-1 Mycobacteroides abscessus subsp. *abscessus* isolated from an Oahu household kitchen sink biofilm and 12-56-S-1-1 M. chimaera isolated from an Oahu household garden soil sample (8). M. avium subsp. *hominissuis* H87 isolated from an indoor sink faucet was also tested (32).

### Microbiome analysis.

Of the 55 soil samples, a subset that included 8 NTM culture-positive and 10 NTM culture-negative soils was subjected to microbiome profiling. DNA was obtained using the PowerSoil DNA isolation kit from MoBio Labs, Inc. (33). Small subunit ribosomal sequencing reads were generated on an Ion Torrent personal genome machine. The V4 region of the 16S rRNA gene was amplified from total extracted DNA using the following primers: 515F, 5′-GTGCCAGCMGCCGCGGTAA-3′, and 806R, 5′-GGACTACHVGGGTWTCTAAT-3′. Sequencing reads were processed through Dada2 (version 1.6.0) to infer sequence variants in R (version 3.4.4) (34, 35). The Dada2 processing pipeline was adjusted to operate on ion torrent semiconductor data by adjusting the homopolymer gap penalty to −1 and increasing the band size parameter to 32 per instructions from the package creators. In addition to the Hawai’i samples, the sequencing run included a no-template control (NTC) to account for spurious amplification during the library preparation. Following sequence variant tabulation with Dada2, counts that remained in the NTC were deducted from the soil samples. The resulting samples had a mean of 22,000 sequence variants per sample with a maximum count of 30,173 and a minimum count of 9,214. Samples were rarified to the minimum count used to establish relative abundance values of sequence variants commonly used in community-level statistics and within the phyloseq R package (version 1.22.3) (36). Taxonomic identification of sequence variants was accomplished in Dada2 using a naive Bayesian classifier against a Dada2-formatted SILVA 128 database (37). Differential abundances of sequence variants by culture status was performed using a negative binomial model through DeSeq2 (version 1.18.1) (38). Genus-level counts of mycobacterium were split into two groups with equal membership using the discretize function of the arules package (version 1.6-1). Visualizations in R were performed with ggplot2 (version 2.2.1) embedded within the phyloseq package. Microbiome data and the code to replicate the figures are freely available on Github at https://github.com/Strong-Lab/NTM_Soil.

### Soil pH and mineralogy.

Soil, saprolite, and fresh rock pH values were measured by adding deionized water to dried material (crushed in the case of fresh rock) until the pore space was saturated and the surface glistened. A standard pH probe and meter were used, and a unique calibration for each sample was generated by measuring pH 4, 7, and 10 buffer solutions.

Minerals were quantified by using a Rigaku MiniFlex 600 X-ray diffractometer (XRD) employing copper radiation and a scintillation detector with a graphite monochromator as a practical rapid screening and characterization tool for complex soil mixtures. Mineral abundances were quantified by standard Rietveld methods embedded in the Rigaku PDXL2 software. Following filtering of columns with sparse information, the resulting matrix contained 11 features for examination, including magnetite, hematite, ilmenite, maghemite, gibbsite, carbonate minerals, quartz, pH, plagioclase, 1:1 clays, and goethite (Table 1).

**TABLE 1:** Information about the minerals used in this study

Mineral	Mineral group	Formula	Expt type
1:1 Clays	1:1 Clays	Al_2_Si_2_O_5_(OH)_4_	*In silico*
Halloysite	1:1 Clay (natural)	Al_2_Si_2_O_5_(OH)_4_	*In vitro*
Kaolinite	1:1 Clay (natural)	Al_2_Si_2_O_5_(OH)_4_	*In vitro*
Gibbsite	Clay (synthetic)	Al(OH)_3_	Both
Magnetite	Fe-Ti oxide/hydroxide	Fe_3_O_4_	Both
Maghemite	Fe-Ti oxide/hydroxide	Fe_2_O_3_	Both
Hematite	Fe-Ti oxide/hydroxide	Fe_2_O_3_	Both
Ilmenite	Fe-Ti oxide/hydroxide	FeTiO_3_	*In silico*
Goetithe	Fe-Ti oxide/hydroxide	FeO(OH)	*In silico*
Calcite/aragonite	Carbonate minerals	CaCO_3_	*In silico*
Plagioclase	Feldspar	NaAlSi_3_O_8_|CaAl_2_Si_2_O_8_	*In silico*
Pyrolusite	Mn oxide (natural)	MnO_2_	*In vitro*
Cryptomelane	K, Mn oxide (natural)	K(Mn)_8_O_16_	*In vitro*
Birnessite	K, Mn oxide (synthetic)	K(Mn)_2_O_4_·1.5H_2_O	*In vitro*
Manganite	Mn oxide/hydroxide (natural)	MnO(OH)	*In vitro*

### Feature correlation analyses.

Feature correlation analyses were used to identify and determine the strength of the correlation between features and response variables. Soil mineralogy was populated using 55 soil samples. The response variable tested was NTM culture status (culture positive or culture negative). Soil characteristics and culture status were imported into a pandas (version 0.20.3) DataFrame object in a Jupyter Notebook (version 4.3.0) using Python (version 3.6). The StandardScaler function from the scikit-learn package (version 0.19.1) was used to normalize soil characteristic percentages within each feature column. The Shapiro-Wilks function from the SciPy package (version 1.0.0) was used to test the normality of each column in both culture status groups. If either group rejected the null hypothesis, a nonparametric Wilcoxon signed-rank test was used to test for significance between NTM culture status. Otherwise, a *t* test with unequal variances was employed to test between the 2 distributions. Feature importance was calculated with the permutation importance function within eli5 (version 0.8.2). Feature values were shuffled in 1,000 permutations, creating an effect of removing the information from a given feature on the performance of a classifier. Thus, features were assigned a mean decrease in accuracy (MDA) signifying how important a feature was to the accuracy of a machine learning model (Fig. S1). MDA features scores were represented in decimal format using Seaborn (version 0.9.0). The balance of samples in our model and the unexplained variance of NTM culture status limits the performance of a classification model and, thus, the overall values of the feature scores (39). The relationship of MDA scores was used to select important features for downstream *in vitro* growth assays. Feature scores changed slightly in each iteration. However, the ordering of importance and significance of the relationships between features remained intact. MDA scores and standard deviations were averaged into a group designated “all remaining features” when not the focus of the soil composition analysis. Feature importance scores only identified soil characteristics useful for accuracy of a machine learning classifier; however, feature importance did not indicate a significant correlation between the abundance of soil features and the outcome variable.

### *In vitro* NTM growth assays in the presence of soil components and sterilized soil samples.

General information for the individual minerals tested in this study is included in Table 1.

The HNL 12-48 soil sample was identified to be rich in kaolinite and free of halloysite and gibbsite. The RAP samples were recovered, by rappelling, from a sea cliff on the northern shoreline of the Kohala peninsula of the Big Island (40). These samples were selected due to the presence of significant quantities of gibbsite and halloysite. Synthetic gibbsite was provided by Barry Bickmore, Brigham Young University (BYU) research collections. Pure birnessite was synthesized by acid titration (41). Crushed hematite was obtained from the BYU research mineral collection. Kaolinite (product no. K1512) and halloysite (product no. 685445) were obtained from Sigma-Aldrich, and maghemite was obtained from U.S. Research Nanomaterials(CAS no. 1309-37-1). After completing a mineral dose-response assay for M. abscessus (Fig. S2) and M. chimaera (Fig. S3), 100 mg/ml of mineral was chosen for *in vitro* growth experiments. Soil samples were autoclaved at 132°C for 15 min, plated on standard Middlebrook 7H10 mycobacterial culture agar (42), and incubated at 37°C for a minimum of 3 days to ensure sample sterility. All particles were suspended to 100 mg/ml in standard mycobacterial culture broth media Middlebrook 7H9 (43) supplemented with 10% albumin-dextrose-catalase (ADC), 2% glycerol, and 0.05% Tween 80. These reagents, both autoclaved and nonsterile, were also characterized with the Rigaku MiniFlex using the CapWow capillary spinner sample holder. Small samples were loaded into 1-mm Kapton tubes and rotated in the X-ray beam, effectively creating a random orientation during analysis.

One milliliter of all suspensions in low-bind microcentrifuge tubes was inoculated with 1 × 10^5^ CFU/ml of 12-45-Sw-A-1 M. abscessus or 5 × 10^5^ CFU/ml of 12-56-S-1-1 M. chimaera or M. avium H87 and incubated on a rotating stand at 37°C (44, 45). The same concentrations of NTM were added to 1 ml 7H9 broth as untreated controls. At the 1-, 24-, 48-, and 96-h time points postinoculation, the cultures were serially diluted in 7H9 broth, and the dilutions were plated in duplicate onto 7H10 agar supplemented with 10% ADC and incubated at 37°C. To determine changes in CFU, the plates were counted 3 days postincubation for M. abscessus and 10 to 14 days postincubation for M. chimaera and M. avium.

### Scanning electron microscopy.

SEM images were obtained for M. abscessus and M. chimaera grown for 48 h in the presence of hematite, gibbsite, birnessite, and untreated controls. Suspensions were filtered through a 0.2-μm Isopore (catalog no. R8MA21491) membrane filter. Next, the samples were fixed with 3% glutaraldehyde in 0.1 M cacodylate buffer (pH 7.3) for 16 h, rinsed with distilled water three times for 10 min, treated with 1% OsO_4_ at 4°C for 16 h, and rinsed again with distilled water. Samples were dehydrated by rinsing for 10 min with ethanol at concentrations of 30, 50, 70, 80, 90, 96, and 100% at 25°C, followed by acetone rinses at 30, 50, and 100% concentrations. Samples were then dried with a critical point dryer, mounted on aluminum SEM stubs with double-sided carbon tape, and coated with a gold-palladium alloy. An FEI Apreo scanning electron microscope at BYU obtained 6-megapixel secondary-electron images in a low vacuum with a 10-kV and 0.1-nA beam.

### *In vitro* data analysis.

Differences in log_10_ CFU/ml between NTM cultures exposed to clays/minerals and unexposed/control cultures were estimated using analysis of variance (ANOVA) models with robust sandwich covariance estimators. Separate models were run for each NTM species (M. abscessus, M. chimaera, and M. avium) at each postexposure time point (1 h, 48 h, 96 h, and, in some experiments, 24 h). Comparisons were made between clay soils, synthetic clays, iron-bearing minerals, and manganese-bearing minerals. ANOVA analyses were performed in R (46) version 3.6.3. Robust covariance estimation was performed using the sandwich package (47) version 2.5-1.

## Supplementary Material

Supplemental file 1
