# Supplementary material for: Assessment of Soil Features on the Growth of Environmental Nontuberculous Mycobacterial Isolates from Hawai'i
Source: Appl Environ Microbiol. 2020 Oct 15;86(21):e00121-20. doi: 10.1128/AEM.00121-20 (PMC7580544; doi:10.1128/AEM.00121-20)
Supplement: Supplemental file 1 [file AEM.00121-20-s0001.pdf]

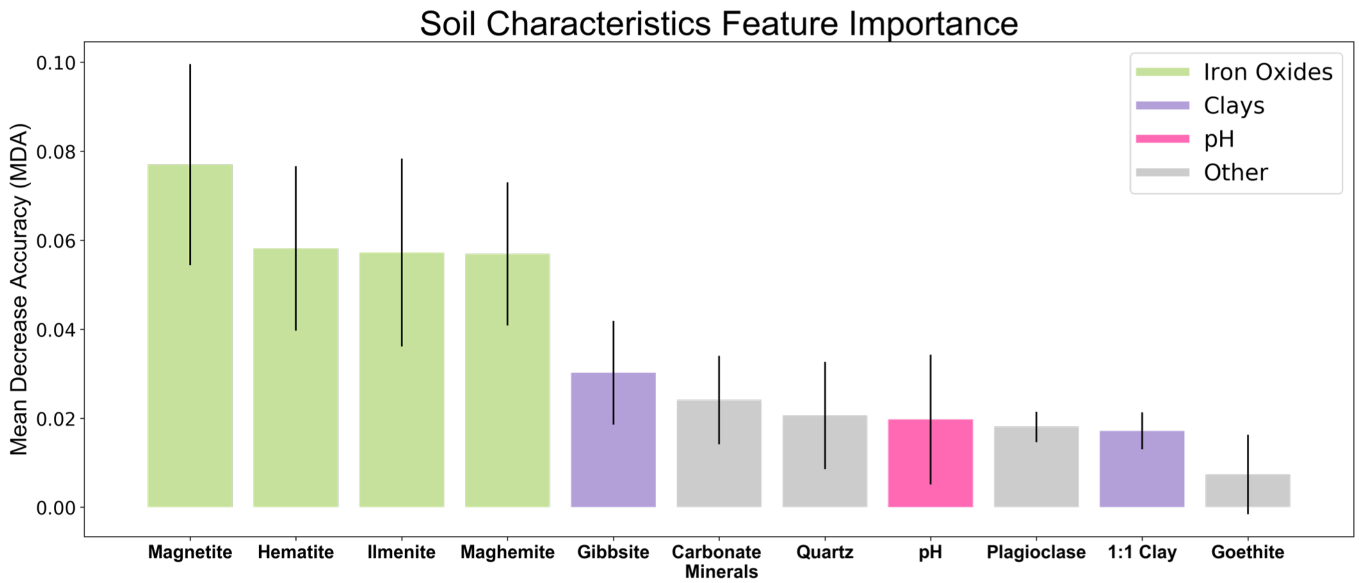

Supplementary Figure 1. Feature importance of all features in the study colored by category in analysis. Feature importance is defined by mean decrease in accuracy (MDA) after 1,000 iterations of a classifier while shuffling the feature values. A higher MDA is associated with an important feature in the model.

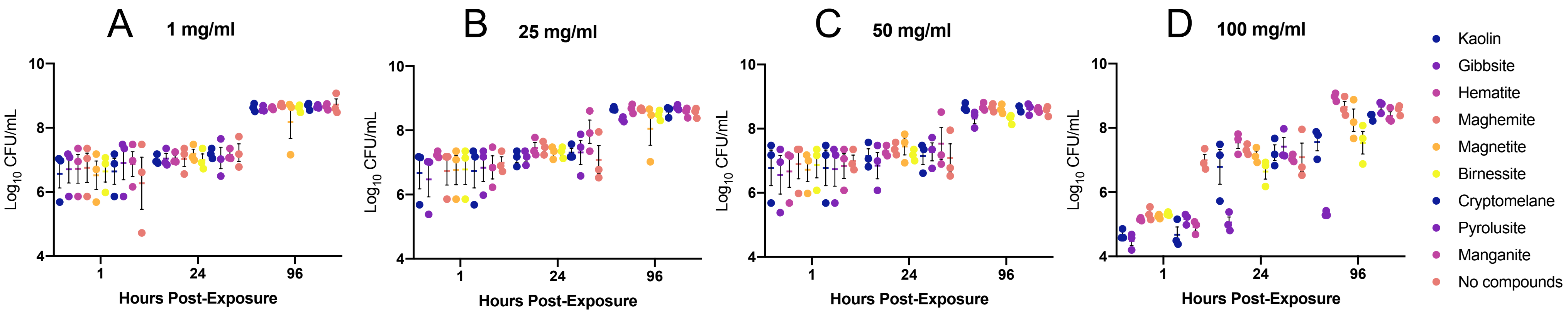

Supplementary Figure 2. *In vitro* growth of *M. abscessus* in the presence of kaolin, gibbsite, hematite, maghemite, magnetite, birnessite, cryptomelane, pyrolusite, and manganite. **A)** 1 mg/ml. **B)** 25 mg/ml. **C)** 50 mg/ml **d)** 100 mg/ml. n = 3 independent experiments.

Supplemental Figure 2
Glickman and Virdi, *et al*

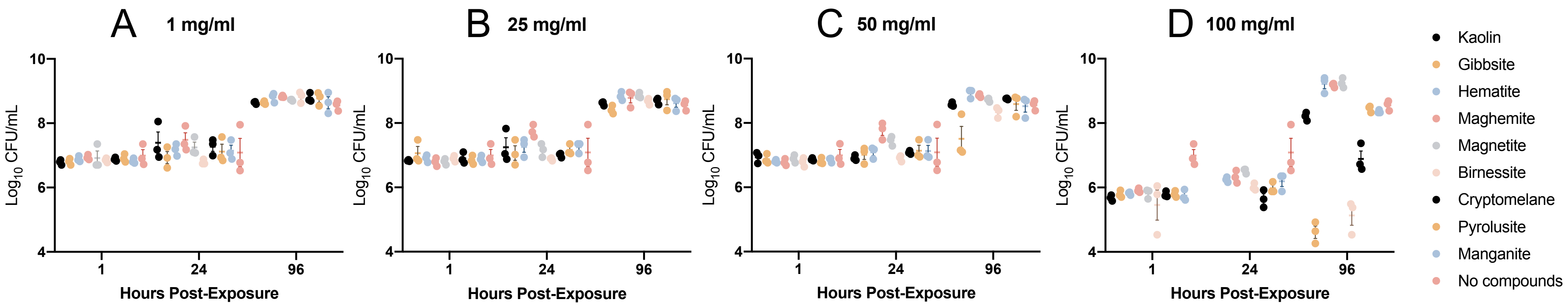

Supplementary Figure 3. *In vitro* growth of *M. chimaera* in the presence of kaolin, gibbsite, hematite, maghemite, magnetite, birnessite, cryptomelane, pyrolusite, and manganite. **A)** 1 mg/ml. **B)** 25 mg/ml. **C)** 50 mg/ml **d)** 100 mg/ml. n = 3 independent experiments.

Supplemental Figure 3
Glickman and Virdi, *et al*

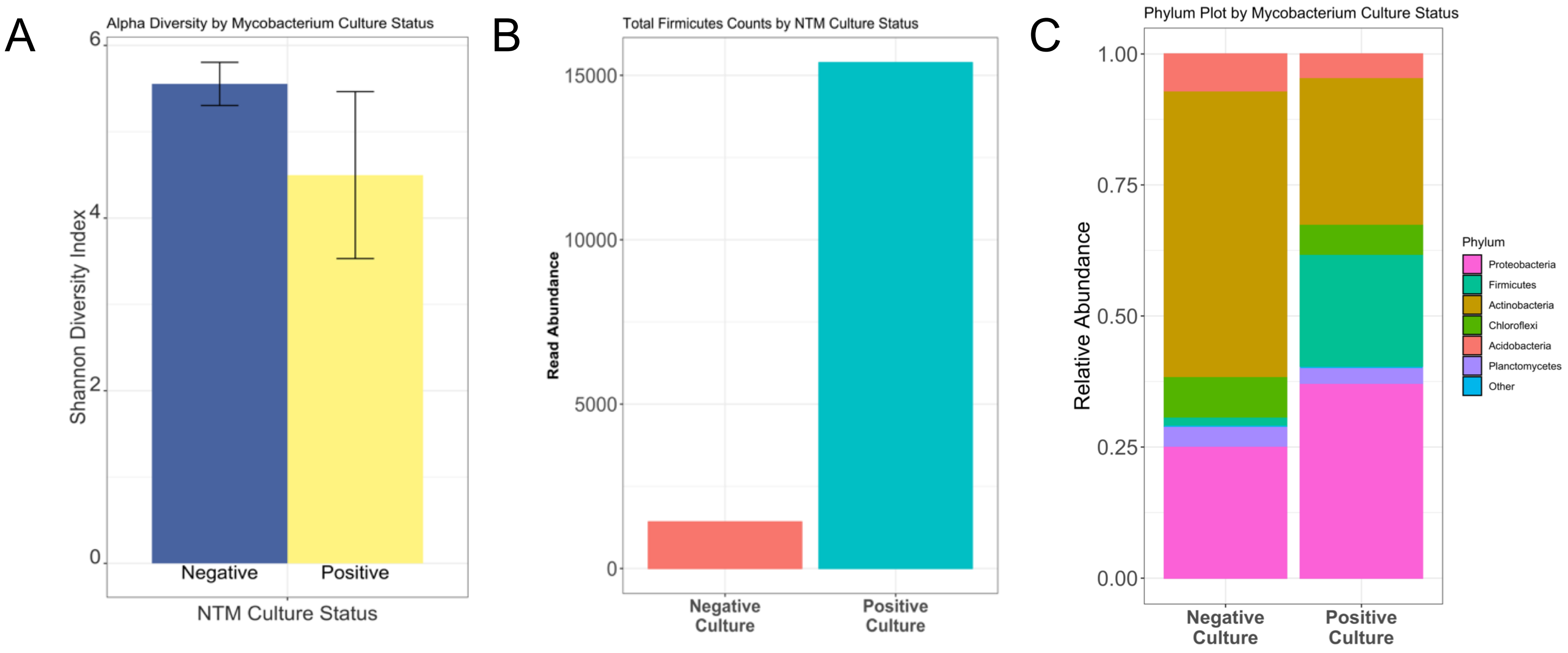

Supplementary Figure 4. A) Shannon alpha diversity values grouped by NTM culture status. NTM culture negative samples averaged a higher alpha diversity value suggesting more species richness than soils that cultured NTM (5.58 ± 0.71 NTM negative vs 4.51 ± 2.25 NTM positive, NS, p = 0.072). **B)** Significantly different phylum *Firmicutes* in microbiome samples stratified by NTM culture status (Log_2FC = 5.109, **, *padj* = 0.0005). **C)** Phylum composition plot in microbiome samples stratified by NTM culture status.

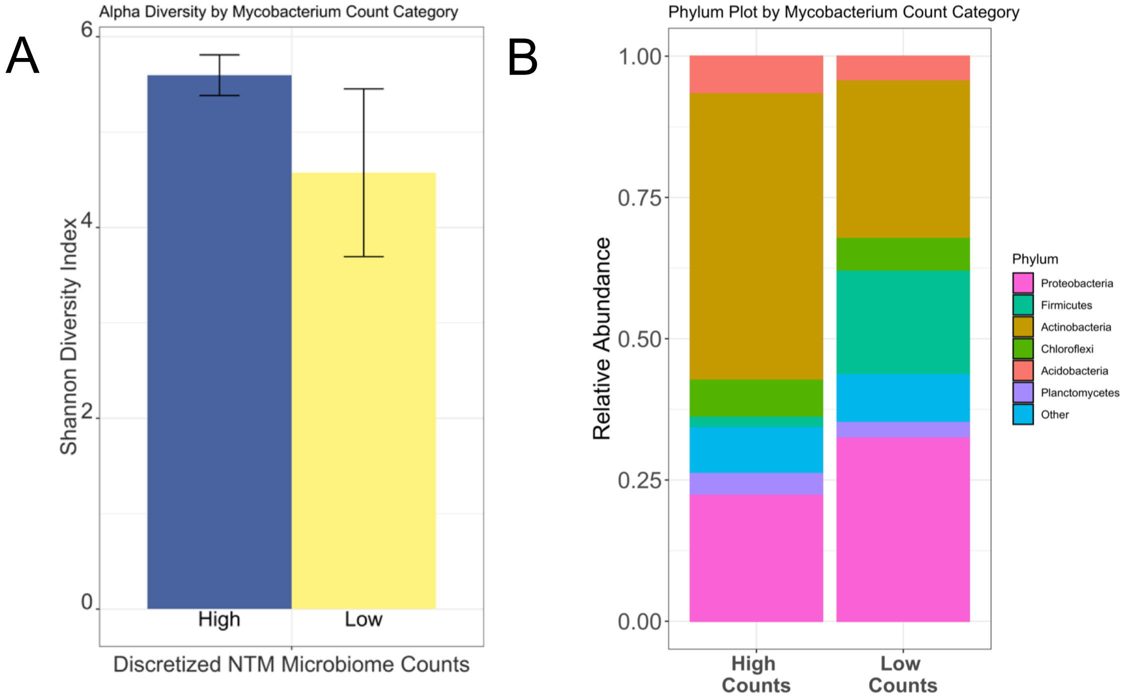

Supplementary Figure 5. A) Shannon alpha diversity values grouped by NTM count categories. On average, samples with higher NTM counts had a greater alpha diversity value than samples with low NTM counts (5.596 ± 0.609 high counts vs 4.574 ± 2.053 low counts, NS, p = 0.054). **B)** Phylum composition plot in microbiome samples stratified by mycobacterium counts.

Supplemental Figure 5
Glickman and Virdi, *et al*

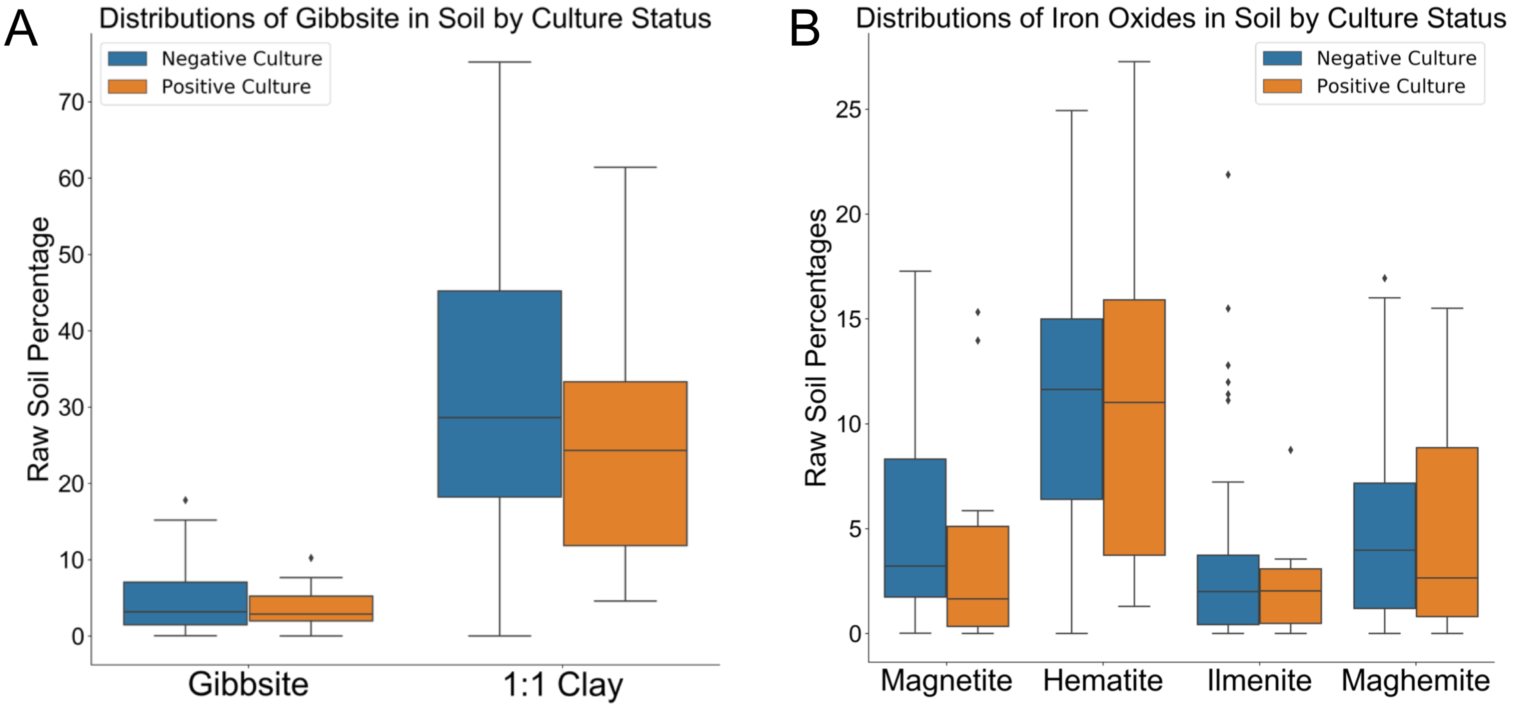

Supplementary Figure 6. **A)** Directionality of raw gibbsite soil concentrations in samples grouped by culture status. The mean abundance of gibbsite is slightly higher in culture negative samples, although the distributions are not significantly different. **B)** Directionality of raw iron oxide mineral soil concentrations in samples grouped by culture status. The mean abundance of all iron oxide minerals is slightly higher in NTM culture negative samples, although the distributions are not significantly different. The mean of magnetite between NTM culture positive and NTM culture negative is the closest to significance (p = 0.109). The upper bound of the variance of hematite in culture positive samples is greater than culture negative samples.

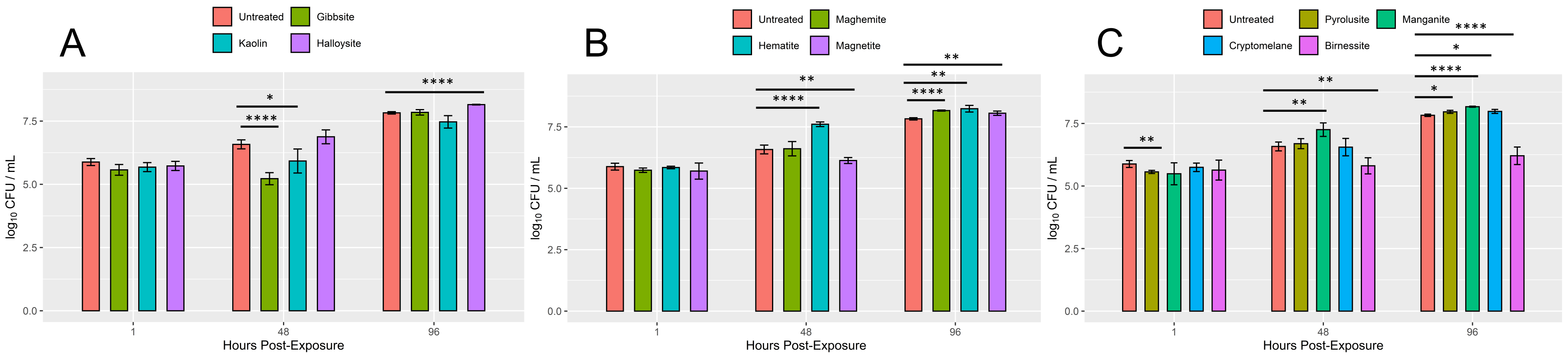

Supplementary Figure 7. *In vitro* growth of *M. avium* in the presence of **A)** synthetic clays gibbsite, kaolin, and halloysite, **B)** synthetic maghemite, magnetite, and hematite, and **C)** synthetic pyrolusite, manganite, cryptomelane, and birnessite. n = 3 independent experiments. * $p < 0.05$, ** $p < 0.01$, *** $p < 0.001$, **** $p < 0.0001$.
